# Insights from the crystal structure of the chicken CREB3 bZIP suggest that members of the CREB3 subfamily transcription factors may be activated in response to oxidative stress

**DOI:** 10.1002/pro.3573

**Published:** 2019-02-06

**Authors:** Keshalini Sabaratnam, Max Renner, Guido Paesen, Karl Harlos, Venugopal Nair, Raymond J. Owens, Jonathan M. Grimes

**Affiliations:** ^1^ Division of Structural Biology University of Oxford Roosevelt Drive, Oxford, OX3 7BN United Kingdom; ^2^ The Pirbright Institute Woking, Guildford, Surrey, GU24 0NF United Kingdom; ^3^ The Research Complex at Harwell Oxfordshire, OX11 0FA United Kingdom; ^4^ Diamond Light Source Limited Oxfordshire, OX11 0DE United Kingdom

**Keywords:** CREB3, bZIP, crystallographic structure, homodimeric bZIP unbound to DNA

## Abstract

cAMP response element binding Protein 3 (CREB3) is an endoplasmic reticulum (ER) membrane‐bound transcription factor, which belongs to the basic leucine zipper (bZIP) superfamily of eukaryotic transcription factors. CREB3 plays a role in the ER‐stress induced unfolded protein response (UPR) and is a multifunctional cellular factor implicated in a number of biological processes including cell proliferation and migration, tumor suppression, and immune‐related gene expression. To gain structural insights into the transcription factor, we determined the crystal structure of the conserved bZIP domain of chicken CREB3 (chCREB3) to a resolution of 3.95 Å. The X‐ray structure provides evidence that chCREB3 can form a stable homodimer. The chCREB3 bZIP has a structured, pre‐formed DNA binding region, even in the absence of DNA, a feature that could potentially enhance both the DNA binding specificity and affinity of chCREB3. Significantly, the homodimeric bZIP possesses an intermolecular disulfide bond that connects equivalent cysteine residues of the parallel helices in the leucine zipper region. This disulfide bond in the hydrophobic core of the bZIP may increase the stability of the homodimer under oxidizing conditions. Moreover, sequence alignment of bZIP sequences from chicken, human, and mouse reveals that only members of the CREB3 subfamily contain this cysteine residue, indicating that it could act as a redox‐sensor. Taken together, these results suggest that the activity of these transcription factors may be redox‐regulated and they may be activated in response to oxidative stress.

## Abstract 2

PDB Code(s): 6IAK

**Abbreviations**
α: Alpha

## Introduction

cAMP response element binding Protein 3 (CREB3) is an endoplasmic reticulum (ER) membrane‐bound transcription factor, which plays a role in the ER‐stress induced unfolded protein response (UPR).^1^ It has been shown to be involved in the ER‐associated degradation (ERAD) pathway, which is regulated by the UPR.^1^, ^2^, ^3^, ^4^ CREB3 also plays a role in Golgi‐stress response,^4^ cell proliferation and migration,^5^, ^6^ tumor suppression,^7^ and inflammatory gene expression^8^, ^9^ and has been shown to be a cellular target of viruses such as Herpes Simplex Virus (HSV), Hepatitis C Virus (HCV), and Human Immunodeficiency Virus (HIV).^7^, ^10^, ^11^

CREB3 belongs to the CREB3 subfamily of the basic leucine zipper (bZIP) superfamily of transcription factors.^12^ bZIP transcription factors bind target DNA sites as homodimers or heterodimers and are characterized by the conserved bZIP domain, composed of a basic region that recognizes a specific DNA sequence and a leucine zipper region that facilitates dimerization.^1^, ^13^ CREB3 is activated by regulated intramembrane proteolysis (RIP) in response to various stimuli including ER‐ and Golgi‐stress.^1^, ^14^ Upon stimulation, the ER‐bound factor is transported to the Golgi apparatus where it is sequentially cleaved by Site 1 protease (S1P) and Site 2 protease (S2P) to liberate the N‐terminal fragment, comprising the transcription activation and bZIP domains. The released N‐terminal fragment translocates to the nucleus to activate transcription of target genes.^1^, ^14^ The sequences recognized by CREB3 include the c‐AMP response element (CRE), ER stress responsive element II (ERSE‐II), and unfolded protein response (UPR) element (UPRE).^1^

Although CREB3 is an important, multifunctional cellular factor, no crystal structures are available for CREB3. We here report the crystal structure of the homodimeric chicken CREB3 (chCREB3) bZIP in the DNA‐free form, determined to a resolution of 3.95 Å. Insights from the structure of the chCREB3 bZIP suggest that members of the CREB3 subfamily of bZIP transcription factors have a putative redox‐sensitive cysteine in the leucine zipper region, hence the activity of these transcription factors may be redox‐regulated.

## Results and Discussion

### *chCREB3 bZIP forms a stable homodimer with a structured DNA‐binding region*

**Figure 1:**
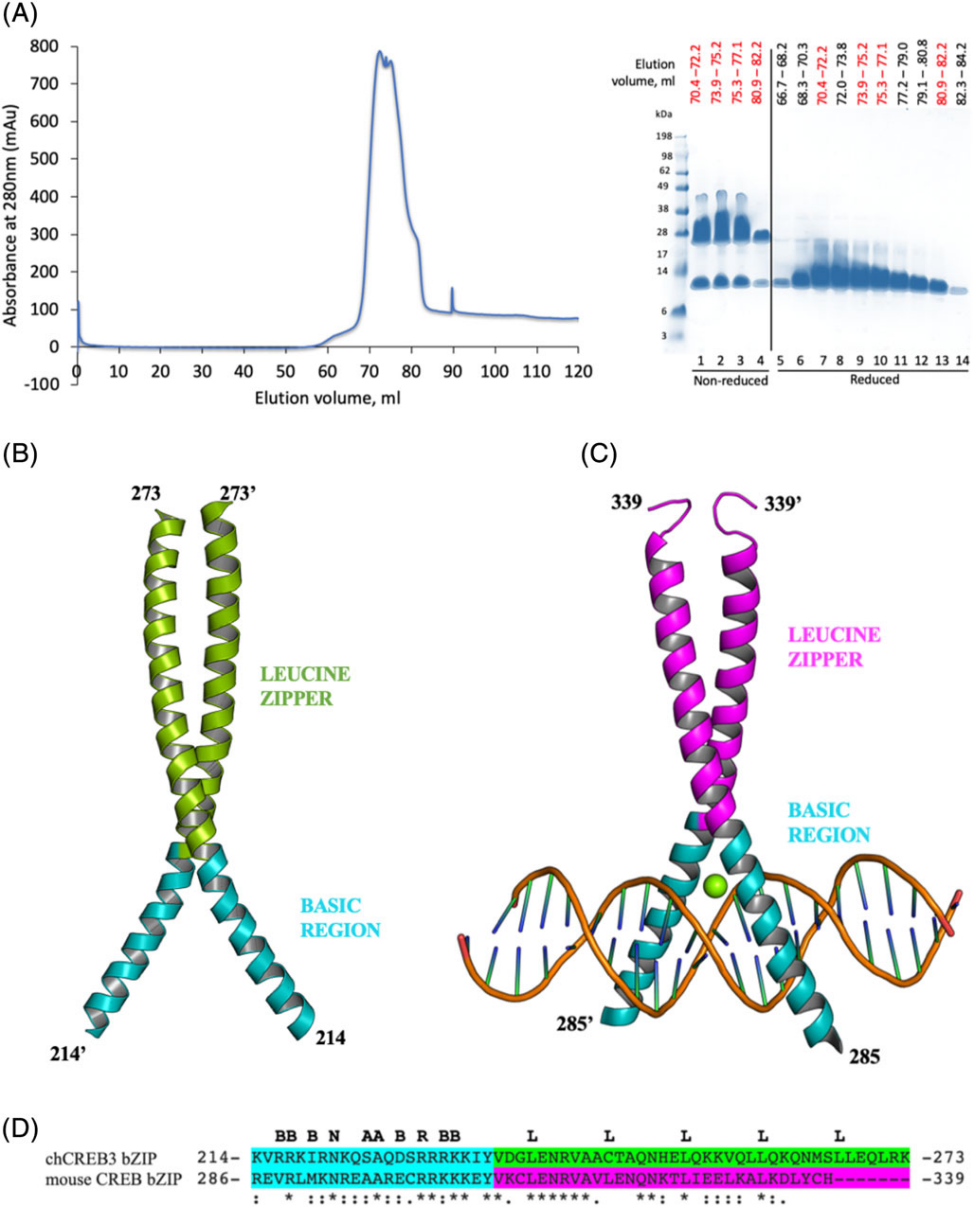
The bZIP domain of chCREB3 forms a stable homodimer with a structured basic region even in the absence of DNA. (A) *Left*: SEC chromatogram of the His tagged chCREB3 bZIP (~11.1 kDa) analyzed on a HiLoad 16/60 Superdex 75 column (GE Healthcare) in 50 m*M* Tris–HCl pH 8, 150 m*M* NaCl. *Right*: selected samples under the peaks were analyzed by non‐reducing SDS‐PAGE (Lanes 1–4). All fractions containing protein were analyzed by reducing SDS‐PAGE (Lanes 5–14). The Coomassie blue‐stained gel is shown here. Samples analyzed by both reducing and non‐reducing SDS‐PAGE are labeled red. Samples were resolved on a 10% Bis‐Tris gel (Invitrogen), which was run in 1× 2‐(N‐morpholino) ethanesulfonic acid (MES) buffer (Invitrogen), alongside the SeeBlue® Plus2 Prestained Protein Standard, Invitrogen. (B) Cartoon representation of the chCREB3 bZIP homodimer showing the structured basic region (cyan) and the leucine zipper region (green). (C) Structure of the CREB bZIP bound to the somatostatin CRE and a hexahydrated Mg(2+) ion (PDB code: 1DH3) (D) Amino acid sequence alignment of chCREB3 (cyan/green) and mouse CREB (cyan/magenta) bZIP sequences. The basic region residues are highlighted in cyan and the leucine zipper residues are highlighted in green or magenta. The consensus sequence is shown above the sequence (B = any basic residue; N = asparagine; A = alanine; R = arginine; L = leucine). An * (asterisk) indicates positions which have a single, fully conserved residue. The bZIP domain of mouse CREB shares 36% amino acid sequence identity with the bZIP domain of chCREB3.

We identified the bZIP domain of chCREB3 (Residues 211–274) using the Uniprot database (http://www.uniprot.org). For crystallization studies, we cloned the region encompassing Residues 206–280 of chCREB3 into the pOPINF expression vector,^15^ which harbors an N‐terminal His6‐3C cleavable tag (MAHHHHHHSS GLEVLFQGP). We expressed the His‐tagged protein (with a predicted molecular weight of ~11.1 kDa) in *Escherichia coli* Rosetta 2(DE3) pLysS cells and purified it by affinity chromatography followed by size exclusion chromatography (SEC). The SEC elution profile displayed three unresolved peaks [Fig. 1(A)]. The bZIP protein was observed in all fractions under the peaks, as determined by denaturing sodium dodecyl sulfate‐polyacrylamide gel electrophoresis (SDS‐PAGE) [Fig. 1(A)]. To determine the composition of the fractions, we assessed selected samples without boiling by non‐reducing SDS‐PAGE. These fractions consisted predominantly of dimeric species [Fig. 1(A)]. These results suggested the presence of a potential inter‐molecular disulfide bridge between chCREB3 bZIP monomers, promoted by the cysteine residue (Cys 246) in the leucine zipper region. We pooled all fractions containing the protein and performed crystallization trials without target DNA and yielded diffraction‐quality crystals that allowed the structure determination.

We determined the crystal structure of the homodimeric chCREB3 bZIP, in the DNA‐free form, to a resolution of 3.95 Å [Fig. 1(B)], by molecular replacement. There are three and a half dimers per asymmetric unit (with one dimer sitting on a crystallographic three‐fold axis). The structure reveals chCREB3 is a stable homodimer even in the absence of DNA. The chCREB3 bZIP forms a continuous α‐helix in which the C‐terminal leucine zipper region forms a parallel coiled‐coil dimerization interface. The N‐terminal basic region of most bZIP transcription factors is largely disordered in the absence of DNA.^16^ However, the chCREB3 bZIP possesses a structured N‐terminal basic region even when unbound to target DNA. This feature has been previously observed in the crystal structure of the heterodimer formed by the bZIP domains of activating transcription factor (ATF) 4 and CCAAT box/enhancer‐binding protein β (C/EBPβ).^17^ In this structure, the basic region of C/EBPβ is disordered, whereas the basic region of ATF4 adopts a stable helical conformation. Podust et al. proposed that the presence of a structured α‐helical DNA‐binding region could act to augment the DNA‐binding specificity of a bZIP transcription factor. The basic region of most bZIP proteins is largely unfolded in the DNA‐unbound state.^16^ Upon binding to DNA, the unfolded basic region becomes α‐helical, bringing into position the DNA‐binding residues that are essential for sequence‐specific interactions with target DNA.^12^ Since structural disorder imparts plasticity, permitting more than one conformation to be adopted,^18^ disorder in the basic region of bZIP domains suggests that the bZIPs can adapt to interact with a broad range of sequences.^17^ In contrast, a well‐defined conformation would restrict interactions with those sequences that are not the specific target site, consequently imposing more stringent binding specificity.^17^ In support of this hypothesis, the crystal structure of the heterodimeric bZIP C/EBPβ:ATF4 unbound to DNA reveals that most of the conserved DNA‐binding residues in the basic region of ATF4 are oriented such that they are in position for specific DNA (CRE) binding. This heterodimeric bZIP was also shown to bind the ATF‐binding site (CRE element) with high affinity, but not the canonical C/EBPβ DNA site (CCAAT box DNA),^17^ implying that the structured DNA binding region of ATF4 might be determining the binding specificity of this heterodimer.

In addition to imposing specificity, possessing an ordered DNA‐binding region could also enhance the DNA‐binding affinity of bZIP proteins. Previous studies have revealed an increase in helical content of bZIP proteins correlates with increased DNA‐binding affinity.^19^, ^20^, ^21^ It has also been proposed that a high intrinsic helicity in the bZIP basic region could increase the overall stability of the bound protein–DNA complex, leading to low dissociation rates.^22^ The converse could be predicted for sequences with low intrinsic helicity.^22^ Taken together, this structural feature of the bZIP suggests that chCREB3 may interact with its specific target site, CRE element, with high affinity. Interestingly, the basic region of the chCREB3 bZIP adopts a similar conformation to the CRE–DNA bound homodimeric bZIP CREB [Fig. 1(B); PDB code: 1DH3].^23^ The bZIP domain of CREB harbors three cysteine residues (Cys300, Cys310, and Cys337), one in the basic region and two in the leucine zipper region. For structural studies, these cysteine residues were mutated to Serine (Ser) to improve protein solubility.^23^, ^24^, ^25^, ^26^, ^27^ These serine mutations, however, have been demonstrated to not alter DNA binding activity of the bZIP.^24^ The root‐mean‐square deviation (RMSD) between the DNA‐unbound chCREB3 bZIP dimers (three copies non‐crystallographic dimers in an asymmetric unit) and the DNA‐bound CREB bZIP dimer was found to range between 0.9 and 1.4 Å. The angular separation of the basic regions in the chCREB3 homodimers (44°, 52°, and 61°) also overlapped the angular separation of the basic regions in the homodimeric bZIP CREB bound to DNA (48.0°).

### *Putative redox‐sensitive cysteine in the leucine zipper region of the bZIP*

**Figure 2:**
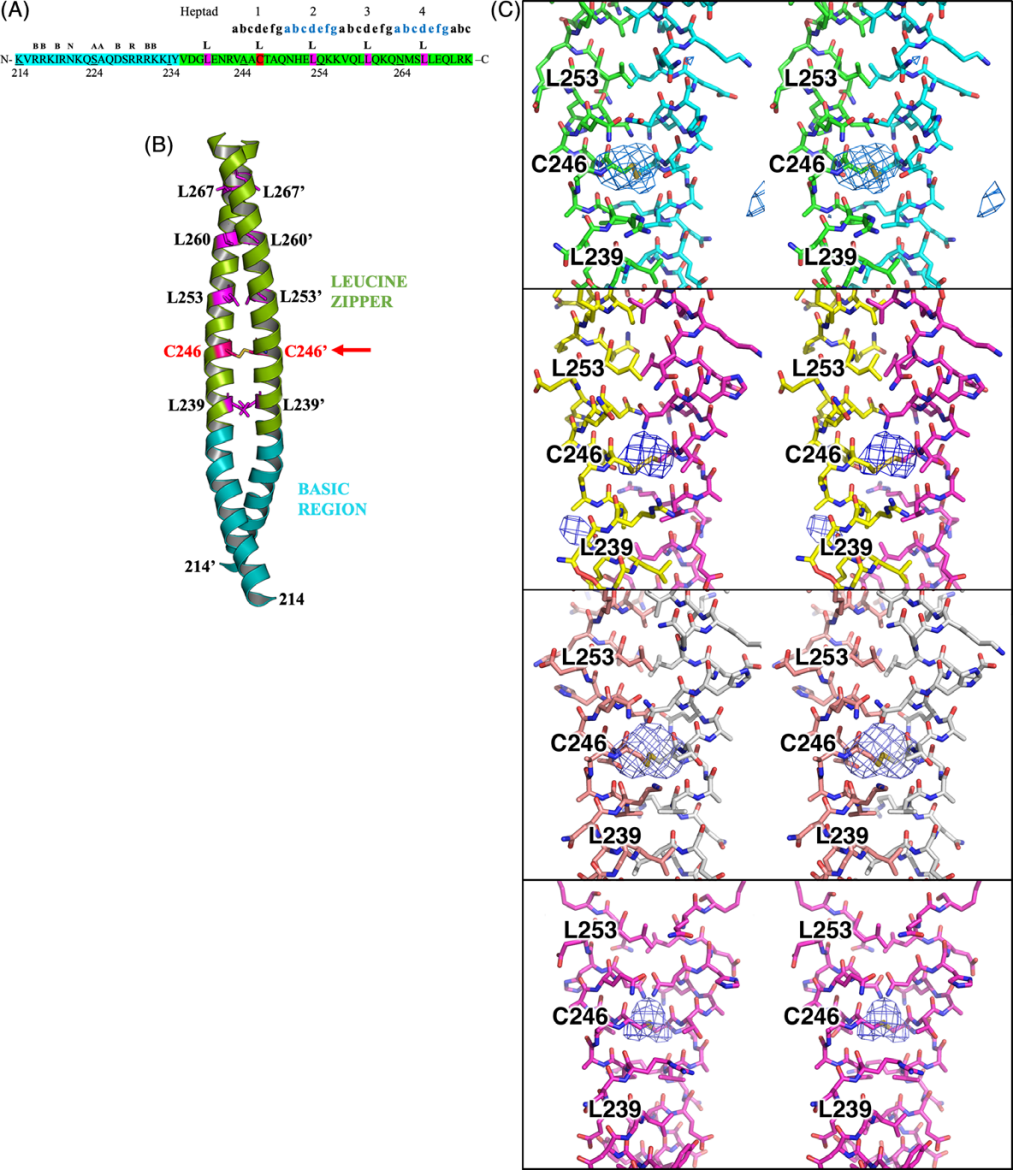
chCREB3 has a disulfide bond in the hydrophobic core of the bZIP. (A) The amino acid sequence of the chCREB3 bZIP composed of heptad repeats with the seven positions of the heptads labeled **a, b, c, d, e, f**, and **g**. The consensus sequence is shown above the sequence (B = any basic residue; N = asparagine; A = alanine; R = arginine; L = leucine). The leucine residues in Position **d** of the heptads are highlighted in pink. The cysteine residue (C246) at Position **d** of the first heptad is highlighted in red. (B) The main chains of the two peptide chains of the chCREB3 bZIP are represented as ribbons. The side chains of the leucine residues and the cysteine participating in the dimer association are represented as stick models. The position of the intermolecular disulfide bond is indicated by a red arrow. (C) Stereo figures of the Fo–Fc difference electron density maps at 3.95 Å resolution, contoured at 3*σ*, for the four chCREB3 bZIP dimers, where Residue Cys 246 has been removed from the model, and crystallographic refinement has been rerun using the initial model. Maps are drawn with a radius of 10 Å centered around Cys 246. The final panel shows the dimer sitting on the crystallographic two‐fold.

A striking feature of the structure of the homodimeric chCREB3 bZIP is the presence of a disulfide bond in the hydrophobic core of the dimer interface (Fig. 2). bZIP family members form dimers through their characteristic leucine zipper helices which consist of seven‐residue repeats of amino acids (denoted **a**–**b**–**c**–**d**–**e**–**f**–**g)**. Hydrophobic residues occur preferentially in the **a** and **d** positions, with a variety of possible amino acids in the **a** positions and usually leucine in the **d** position.^28^ Hydrophobic interactions by leucine and other hydrophobic amino acids in **a** and **d** positions in the helix form the hydrophobic core of the bZIP dimer, facilitating dimerization.^28^ Interestingly, a cysteine residue (Cys 246) occupies the **d** position of the first heptad of the chCREB3 bZIP and forms a disulfide bond with the cysteine from its dimeric partner. Difference electron density maps, where Cys 246 has been omitted from the refinement, show strong density features for the disulfide [Fig. 2(C)]. Mutational studies have demonstrated that amino acids in Positions **a** and **d** regulate leucine zipper oligomerization, dimerization stability, and specificity.^28^ This cysteine residue replacing the consensus leucine at this position, by forming a covalent bond, likely enhances the dimer stability.

This is the first time a disulfide bond has been observed in a crystal structure of a bZIP domain, although previous studies have reported evidence for the presence of an intermolecular disulfide bond in the leucine zipper region of ATF4 and ATF5 bZIP homodimers. These proteins possess a cysteine residue at Position **a** of the first heptad in the bZIP region. Circular dichroism (CD) and nuclear magnetic resonance (NMR) studies of ATF4 and ATF5 bZIPs have indicated that in the absence of DNA, these bZIPs are only partially structured, are predominantly monomeric, and are not capable of forming stable homodimers, even in the presence of a disulfide bond.^17^, ^29^ In contrast, our study reveals the bZIP domain of chCREB3 that has a cysteine residue at the first **d** position is able to form a stable homodimer, even in the absence of DNA. Taken together, these results suggest that an intermolecular disulfide bond at the first **d** position may play a role in promoting homodimerization and enhancing dimer stability. Moreover, Podust et al.^17^ and Ciaccio and Laurence^29^ reported that the disulfide bond formation increased the α‐helical content of the bZIP domains of ATF4 and ATF5 and suggested that an intermolecular disulfide bond could act to stabilize a coiled‐coil. Based on these findings, this disulfide bond in the chCREB3 bZIP may be important for extending the stabilizing α‐helical conformation from the leucine zipper region to the basic region.^30^, ^31^, ^32^, ^33^, ^34^, ^35^, ^36^, ^37^

Multiple sequence alignment analysis of bZIP domain sequences of chicken, human, and mouse bZIP transcription factors reveals that only members of the CREB3 subfamily (CREB3, CREB3L2, and CREB3L3 of the chicken CREB3 subfamily, CREB3L2, CREB3L3, and CREB3L4 of the human CREB3 subfamily and CREB3L2, CREB3L3, and CREB3L4 of the mouse CREB3 subfamily) contain a cysteine residue at Position **d** of the first heptad (Fig. S1). These results suggest that this cysteine, which is unique for the CREB3 subfamily, could act as a redox‐sensor; hence, the activity of these transcription factors may be redox‐regulated. Several transcription factors have been reported to contain redox‐sensitive cysteine residues at their DNA‐binding sites, which affect their DNA‐binding ability.^38^ In most cases, oxidation of critical cysteine residues negatively regulates the DNA‐binding activity of these transcription factors.^38^ Examples of factors whose DNA‐binding activity is negatively regulated by oxidation include the heterodimeric bZIP transcription factor Fos/Jun, the nuclear factor kappa B (NF‐*κ*B), and hypoxia‐inducible factor‐1α (HIF‐1α).^39^, ^40^, ^41^, ^42^ In contrast, an example of a transcription factor whose DNA‐binding activity is up‐regulated by oxidative stress is HoxB5, a member of the Hox family of proteins.^40^, ^43^ Oxidative conditions trigger the dimerization of HoxB5, a structural reorganization required for the cooperative binding of this factor to tandem DNA target sites.^40^, ^43^ A critical cysteine residue (Cys 232) located in the conserved homeobox encoded DNA‐binding domain is essential for this redox regulation.^40^, ^43^ The critical cysteine of the chCREB3 bZIP is located downstream of the DNA‐binding region and takes part in dimerization. Hence, as observed for HoxB5, the oxidation of this cysteine is likely to upregulate the DNA‐binding activity of chCREB3 by promoting dimerization and structural changes required for DNA binding.

Based on the results of this study, we hypothesize that chCREB3 and other members of the CREB3 subfamily may be able to form stable or weakly associated homodimers depending on the oxidation state of the cell. During non‐oxidizing conditions, lacking a disulfide bond, these bZIP factors may form weakly associated homodimers in the absence of DNA. We predict these weakly bound dimers to possess a largely unfolded DNA‐binding region and the flexibility may enable them to bind a range of sequences, although the interaction with these sites may be weak. Under oxidizing conditions, the formation of a disulfide bond may enhance dimer stability and induce structural changes to the basic DNA‐binding region of these bZIP factors, influencing target DNA interactions. These structural changes may augment DNA‐binding specificity and affinity and could act to potentiate the activity of these factors. Further experiments will need to be performed to determine if the disulfide bond is indeed essential for promoting homodimerization stability and whether it is required for inducing structural changes to the basic DNA‐binding region of the bZIP domain of chCREB3.

## Materials and Methods

### *Protein expression and purification*

The gene encoding the bZIP domain of chCREB3 (Residues 206–280) was cloned into the pOPINF expression vector,^15^ which harbors an *N‐terminal His*6*‐3C* cleavable tag (MAHHHHHHSS GLEVLFQGP). For protein expression, competent *E. coli* Rosetta 2(DE3) pLysS cells were transformed with the expression plasmid. Cultures were grown to an OD_600_ of 0.5–0.6 at 37°C, induced with 0.5 m*M* isopropyl β‐d‐1‐thiogalactopyranoside (IPTG) and grown for 3 h at 37°C. For protein extraction, the pellets were resuspended in 50 m*M* Tris‐hydrochloride (HCl) pH 8, 150 m*M* NaCl, 0.1% Tween 20, 1× Roche Complete EDTA free protease inhibitors and lysed by sonication. His‐tagged protein was purified using Nickel–nitrilotriacetic acid (Ni‐NTA) agarose and eluted with buffer containing 50 m*M* Tris–HCl pH 8, 150 m*M* NaCl, and 300 m*M* Imidazole. The protein was further purified by size exclusion chromatography (SEC) using a Superdex 75 column (GE Healthcare) in 50 m*M* Tris–HCl, pH 8, 150 m*M* NaCl. All fractions containing the bZIP domain of chCREB3 were pooled and concentrated and used for crystallization.

### *Protein analysis*

For denaturing SDS‐PAGE, protein samples were mixed with 4× SDS sample loading buffer (250 m*M* Tris pH 6.8, 8% w/v SDS, 0.02% w/v bromophenol blue, 40% v/v glycerol, 8% β‐mercaptoethanol (BME)], heated to 99°C for 10 min, and spun down before loading. Samples were resolved on a 10% Bis‐Tris gels (Invitrogen), which were run in 1× MES buffer (Invitrogen) using the NuPage Novex gel system (Invitrogen). Ten microliter of samples were loaded per well alongside the SeeBlue® Plus2 Prestained Protein Standard (3–198 kDa), Invitrogen.

### *Crystallization, data collection, and structure determination*

The bZIP domain of chCREB3 was crystallized using the sitting‐drop vapor‐diffusion method in 96‐well plates (CrystalQuick, Greiner Bio‐One, Germany).^44^ Drops were prepared using 100 nL protein solution mixed with 100 nL reservoir solution and were equilibrated against 100 μL precipitant solution. The concentration of the protein was 44 mg mL^−1^. Clusters consisting of thin, rod‐shaped crystals were obtained at 4°C with the reservoir condition 50% v/v 2‐methyl‐2,4‐pentanediol (MPD), 100 m*M* Tri‐sodium citrate pH 5.6, 10 m*M* magnesium chloride (MORPHEUS screen).^45^ After optimization, diffraction‐quality crystals were obtained from a drop containing 43% v/v 2‐methyl‐2,4‐pentanediol (MPD), 100 m*M* tri‐sodium citrate pH 5.6, 10 m*M* magnesium chloride. For data collection, crystals of the chCREB3 bZIP were dragged through a drop of the cryoprotectant (MPD)‐containing reservoir solution and cooled to liquid nitrogen temperature. Diffraction data were collected at 100 K at Diamond Light Source, beamline I04. A data set extending to a resolution of 3.95 Å was collected on a single crystal of the chCREB3 bZIP covering a 180° of rotation range. The data were auto‐processed using Xia2 (Winter et al., 2010). Data processing statistics are summarized in Table 1. The crystals belonged to the base‐centered orthorhombic Space group C222, with unit cell parameters *a* = 137.90, *b* = 167.06, and *c* = 115.46 Å. The crystals contained seven copies of the chCREB3 bZIP monomer per asymmetric unit, arranged as three and half dimers, with one dimer sitting on crystallographic two‐fold axis. Seven copies of the bZIP monomer give a solvent content of approximately 85%.

**Table 1:** Data collection and Refinement statistics

*Data collection*	
Wavelength (Å)	0.98
Space group	C 2 2 2
Unit cell parameters	
*a*, *b*, *c* (Å)	137.90, 167.06, 115.46
*α*, *β*, *γ* (°)	90.00, 90.00, 90.00
Resolution (Å)	51.63–3.95 (4.09–3.95)^a^
CC [1/2]	0.93 (0.76)
*R*_meas_	0.30 (1.80)
Total no. of reflections	77770 (5573)
Unique reflections	12046 (887)
Mean [(*I*)/*σ*(*I*)]	3.30 (1.00)
Completeness (%)	100.00 (100.00)
Multiplicity	6.5 (6.3)
*Refinement*	
Number of reflections	73,881 (5294)
Resolution (Å)	51.63–3.95 (4.09–3.95)
*R*_work_	0.26
*R*_Free_	0.30
No. of non‐H atoms	
Protein	3563
Mean *B*‐value (Å^2)^	
Protein	184.88
RMSD from ideal values	
Bond lengths (Å)	0.020
Bond angles (°)	2.18
Ramachandran statistics (%)	
Preferred regions	99
Outliers	1

RMSD, root mean square deviation from ideal geometry; CC (1/2), cross‐correlation between random half‐datasets. *R*_free_ was calculated for a 5% subset of reflections.

a Values in parentheses correspond to those of the highest resolution shell.

The structure of the bZIP domain of chCREB3 was solved by molecular replacement with the program PHASER^46^ using a dimer extracted from the CREB bZIP–CRE complex structure (PDB code: 1DH3) after the deletion of the bound duplex DNA. Three dimers were found using PHASER, and the final monomer identified from visualization of 2Fo–Fc and Fo–Fc electron density maps. The bZIP domain of mouse CREB has 36% amino acid sequence identity to the bZIP domain of chCREB3. The structure was refined by iterative rounds of manual model building in COOT^47^ and using BUSTER.^48^ Refinement resulted in *R*_work_ and *R*_free_ values of 0.26 and 0.30, respectively. Refinement statistics are summarized in Table 1. The geometry of the final model was validated with MOLPROBITY.^49^ Protein coordinates have been deposited in the Protein Data Bank (PDB entry 6IAK). Figures were generated using PyMOL (DeLano WL 2002).

### *Sequence and structural alignments*

bZIP sequences were obtained from the Uniprot database (http://www.uniprot.org/). Multiple sequence alignments were performed using Clustal Omega.^50^ Structural alignments were performed using PyMOL (DeLano WL 2002).

## Supplementary Material

Amino acid sequence alignment of bZIP sequences (Fig. S1). Stereo image of the unit cell and the contents of the asymmetric units. The bZIP domain molecules in each asymmetric unit are coloured identically (Fig S2).

## Funding information

Wellcome Trust, Medical Research Council, Biotechnology and Biological Sciences Research Council; Pirbright Institute; University of Oxford.

## Supporting information

Figure S1 The bZIP regions of chicken (A), human (B), and mouse (C) bZIP transcription factors were aligned using Clustal. The consensus sequence is shown above the sequence alignment (B = any basic residue; N = asparagine; A = alanine; R = arginine; L = leucine). As described in C. Vinson et al. (2002), proteins were placed into 12 groups based on predicted dimerization properties. The basic and leucine zipper regions are highlighted in cyan and green, respectively. Within the leucine zipper region, leucine residues at position d of the heptads are highlighted in magenta; cysteine residues at position d of the heptads are highlighted in red; any residue other than leucine or cysteine at position d of the heptads is highlighted in gray; cysteine residues at any position other than d are highlighted in yellow; and asparagine residues that occupy position a of the second and fourth heptads are highlighted.

Figure S2 Stereo image of the unit cell and the contents of the asymmetric units. The bZIP domain molecules in each asymmetric unit are colored identically.

## References

[1] Chan C‐P , Kok K‐H , Jin D‐Y (2011) CREB3 subfamily transcription factors are not created equal: recent insights from global analyses and animal models. Cell Biosci 1:6. 21711675 10.1186/2045-3701-1-6 PMC3116243

[2] DenBoer LM , Hardy‐Smith PW , Hogan MR , Cockram GP , Audas TE , Lu R (2005) Luman is capable of binding and activating transcription from the unfolded protein response element. Biochem Biophys Res Commun 331:113–119. 15845366 10.1016/j.bbrc.2005.03.141

[3] Liang G , Audas TE , Li Y , Cockram GP , Dean JD , Martyn AC , Kokame K , Lu R (2006) Luman/CREB3 induces transcription of the endoplasmic reticulum (ER) stress response protein herp through an ER stress response element. Mol Cell Biol 26:7999–8010. 16940180 10.1128/MCB.01046-06 PMC1636730

[4] Reiling JH , Olive AJ , Sanyal S , Carette JE , Brummelkamp TR , Ploegh HL , Starnbach MN , Sabatini DM (2013) A CREB3–ARF4 signalling pathway mediates the response to Golgi stress and susceptibility to pathogens. Nat Cell Biol 15:1473–1485. 24185178 10.1038/ncb2865 PMC3965854

[5] Jang S‐W , Kim YS , Kim YR , Sung HJ , Ko J (2007) Regulation of human LZIP expression by NF‐κB and its involvement in monocyte cell migration induced by Lkn‐1. J Biol Chem 282:11092–11100. 17296613 10.1074/jbc.M607962200

[6] Kim H‐C , Choi K‐C , Choi H‐K , Kang H‐B , Kim M‐J , Lee Y‐H , Lee O‐H , Lee J , Kim YJ , Jun W (2010) HDAC3 selectively represses CREB3‐mediated transcription and migration of metastatic breast cancer cells. Cell Mol Life Sci 67:3499–3510. 20473547 10.1007/s00018-010-0388-5 PMC11115716

[7] Jin D‐Y , Wang H‐L , Zhou Y , Chun ACS , Kibler KV , Hou Y‐D , Kung H , Jeang K‐T (2000) Hepatitis C virus core protein‐induced loss of LZIP function correlates with cellular transformation. EMBO J 19:729–740. 10675342 10.1093/emboj/19.4.729 PMC305611

[8] Sung HJ , Kim YS , Kang H , Ko J (2008) Human LZIP induces monocyte CC chemokine receptor 2 expression leading to enhancement of monocyte chemoattractant protein 1/CCL2‐induced cell migration. Exp Mol Med 40:332–338. 18587271 10.3858/emm.2008.40.3.332 PMC2679297

[9] Eleveld‐Trancikova D , Sanecka A , van Hout‐Kuijer MA , Looman MW , Hendriks IA , Jansen BJ , Adema GJ (2010) DC‐STAMP interacts with ER‐resident transcription factor LUMAN which becomes activated during DC maturation. Mol Immunol 47:1963–1973. 20546900 10.1016/j.molimm.2010.04.019

[10] Lu R , Misra V (2000) Potential role for luman, the cellular homologue of Herpes simplex virus VP16 (α gene trans‐inducing factor), in Herpes virus latency. J Virol 74:934–943. 10623756 10.1128/jvi.74.2.934-943.2000 PMC111614

[11] Blot G , Lopez‐Vergès S , Treand C , Kubat NJ , Delcroix‐Genête D , Emiliani S , Benarous R , Berlioz‐Torrent C (2006) Luman, a new partner of HIV‐1 TMgp41, interferes with Tat‐mediated transcription of the HIV‐1 LTR. J Mol Biol 364:1034–1047. 17054986 10.1016/j.jmb.2006.09.080

[12] Miotto B , Struhl K (2006) Differential gene regulation by selective association of transcriptional coactivators and bZIP DNA‐binding domains. Mol Cell Biol 26:5969–5982. 16880509 10.1128/MCB.00696-06 PMC1592802

[13] Zhang Y , Zhou J , Wang L (2013) Mini review roles of the bZIP gene family in rice. Genet Mol Res 13:3025–3036. 10.4238/2014.April.16.11 24782137

[14] Taniguchi M , Yoshida H (2017) TFE3, HSP47, and CREB3 pathways of the mammalian golgi stress response. Cell Struct Funct 42:27–36. 28179603 10.1247/csf.16023

[15] Berrow NS , Alderton D , Sainsbury S , Nettleship J , Assenberg R , Rahman N , Stuart DI , Owens RJ (2007) A versatile ligation‐independent cloning method suitable for high‐throughput expression screening applications. Nucleic Acids Res 35:e45–e45. 17317681 10.1093/nar/gkm047 PMC1874605

[16] Hollenbeck JJ , McClain DL , Oakley MG (2002) The role of helix stabilizing residues in GCN4 basic region folding and DNA binding. Protein Sci 11:2740–2747. 12381856 10.1110/ps.0211102 PMC2373721

[17] Podust LM , Krezel AM , Kim Y (2001) Crystal structure of the CCAAT box/enhancer‐binding protein β activating transcription factor‐4 basic leucine zipper heterodimer in the absence of DNA. J Biol Chem 276:505–513. 11018027 10.1074/jbc.M005594200

[18] Babu MM , van der Lee R , de Groot NS , Gsponer J (2011) Intrinsically disordered proteins: regulation and disease. Curr Opin Struct Biol 21:432–440. 21514144 10.1016/j.sbi.2011.03.011

[19] Szilák L , Moitra J , Krylov D , Vinson C (1997) Phosphorylation destabilizes α‐helices. Nat Struct Biol 4:112–114. 9033589 10.1038/nsb0297-112

[20] Szilák L , Moitra J , Vinson C (1997) Design of a leucine zipper coiled coil stabilized 1.4 kcal mol^−1^ by phosphorylation of a serine in the e position. Protein Sci 6:1273–1283. 9194187 10.1002/pro.5560060615 PMC2143729

[21] Woolley GA , Jaikaran AS , Berezovski M , Calarco JP , Krylov SN , Smart OS , Kumita JR (2006) Reversible photocontrol of DNA binding by a designed GCN4‐bZIP protein. Biochemistry 45:6075–6084. 16681380 10.1021/bi060142r

[22] Das RK , Crick SL , Pappu RV (2012) N‐terminal segments modulate the α‐helical propensities of the intrinsically disordered basic regions of bZIP proteins. J Mol Biol 416:287–299. 22226835 10.1016/j.jmb.2011.12.043

[23] Schumacher MA , Goodman RH , Brennan RG (2000) The structure of a CREB bZIP· somatostatin CRE complex reveals the basis for selective dimerization and divalent cation‐enhanced DNA binding. J Biol Chem 275:35242–35247. 10952992 10.1074/jbc.M007293200

[24] Goren I , Tavor E , Goldblum A , Honigman A (2001) Two cysteine residues in the DNA‐binding domain of CREB control binding to CRE and CREB‐mediated gene expression1. J Mol Biol 313:695–709. 11697898 10.1006/jmbi.2001.5064

[25] Luo Q , Viste K , Urday‐Zaa JC , Kumar GS , Tsai W‐W , Talai A , Mayo KE , Montminy M , Radhakrishnan I (2012) Mechanism of CREB recognition and coactivation by the CREB‐regulated transcriptional coactivator CRTC2. Proc Natl Acad Sci U S A 109:20865–20870. 23213254 10.1073/pnas.1219028109 PMC3529076

[26] Cardinaux J‐R , Notis JC , Zhang Q , Vo N , Craig JC , Fass DM , Brennan RG , Goodman RH (2000) Recruitment of CREB binding protein is sufficient for CREB‐mediated gene activation. Mol Cell Biol 20:1546–1552. 10669732 10.1128/mcb.20.5.1546-1552.2000 PMC85336

[27] Richards JP , Bächinger HP , Goodman RH , Brennan RG (1996) Analysis of the structural properties of cAMP‐responsive element‐binding protein (CREB) and phosphorylated CREB. J Biol Chem 271:13716–13723. 8662719 10.1074/jbc.271.23.13716

[28] Vinson C , Myakishev M , Acharya A , Mir AA , Moll JR , Bonovich M (2002) Classification of human B‐ZIP proteins based on dimerization properties. Mol Cell Biol 22:6321–6335. 12192032 10.1128/MCB.22.18.6321-6335.2002 PMC135624

[29] Ciaccio NA , Laurence JS (2009) Effects of disulfide bond formation and protein helicity on the aggregation of activating transcription factor 5 (ATF5). Mol Pharma 6:1205–1215. 10.1021/mp900058t PMC3414431 19435374

[30] Kammerer RA (1997) α‐Helical coiled‐coil oligomerization domains in extracellular proteins. Matrix Biol 15:555–565. 9138288 10.1016/s0945-053x(97)90031-7

[31] Mason JM , Arndt KM (2004) Coiled coil domains: stability, specificity, and biological implications. Chembiochem 5:170–176. 14760737 10.1002/cbic.200300781

[32] Krylov D , Mikhailenko I , Vinson C (1994) A thermodynamic scale for leucine zipper stability and dimerization specificity: e and g interhelical interactions. EMBO J 13:2849–2861. 8026470 10.1002/j.1460-2075.1994.tb06579.x PMC395166

[33] Croasdale R , Ivins FJ , Muskett F , Daviter T , Scott DJ , Hardy T , Smerdon SJ , Fry AM , Pfuhl M (2011) An undecided coiled‐coil: the leucine zipper of NEK2 kinase exhibits atypical conformational exchange dynamics. J Biol Chem 286:27537–27547. 21669869 10.1074/jbc.M110.196972 PMC3149346

[34] Welbourn S , Kao S , Du Pont KE , Andrew AJ , Berndsen CE , Strebel K (2015) Positioning of cysteine residues within the N‐terminal portion of the BST‐2/tetherin ectodomain is important for functional dimerization of BST‐2. J Biol Chem 290:3740–3751. 25525265 10.1074/jbc.M114.617639 PMC4319038

[35] Moitra J , Szilák L , Krylov D , Vinson C (1997) Leucine is the most stabilizing aliphatic amino acid in the d position of a dimeric leucine zipper coiled coil. Biochemistry 36:12567–12573. 9376362 10.1021/bi971424h

[36] Cranz S , Berger C , Baici A , Jelesarov I , Bosshard HR (2004) Monomeric and dimeric bZIP transcription factor GCN4 bind at the same rate to their target DNA site. Biochemistry 43:718–727. 14730976 10.1021/bi0355793

[37] Moll JR , Ruvinov SB , Pastan I , Vinson C (2001) Designed heterodimerizing leucine zippers with a ranger of pIs and stabilities up to 10− 15 M. Protein Sci 10:649–655. 11344333 10.1110/ps.39401 PMC2374140

[38] Trachootham D , Lu W , Ogasawara MA , Valle NR‐D , Huang P (2008) Redox regulation of cell survival. Antioxid Redox Signal 10:1343–1374. 18522489 10.1089/ars.2007.1957 PMC2932530

[39] Amoutzias GD , Bornberg‐Bauer E , Oliver SG , Robertson DL (2006) Reduction/oxidation‐phosphorylation control of DNA binding in the bZIP dimerization network. BMC Genomics 7:107. 16674813 10.1186/1471-2164-7-107 PMC1479340

[40] Arrigo A‐P (1999) Gene expression and the thiol redox state. Free Rad Biol Med 27:936–944. 10569626 10.1016/s0891-5849(99)00175-6

[41] Morel Y , Barouki R (1999) Repression of gene expression by oxidative stress. Biochem J 342:481–496. 10477257 PMC1220487

[42] Bannister A , Cook A , Kouzarides T (1991) In vitro DNA binding activity of Fos/Jun and BZLF1 but not C/EBP is affected by redox changes. Oncogene 6:1243–1250. 1907361

[43] Galang CK , Hauser CA (1993) Cooperative DNA binding of the human HoxB5 (Hox‐2.1) protein is under redox regulation in vitro. Mol Cell Biol 13:4609–4617. 8101633 10.1128/mcb.13.8.4609-4617.1993 PMC360087

[44] Walter TS , Diprose JM , Mayo CJ , Siebold C , Pickford MG , Carter L , Sutton GC , Berrow NS , Brown J , Berry IM , Stewart‐Jones GBE , Grimes JM , Stammers DK , Esnouf RM , Jones EY , Owens RJ , Stuart DI , Harlos K (2005) A procedure for setting up high‐throughput nanolitre crystallization experiments. Crystallization workflow for initial screening, automated storage, imaging and optimization. Acta Cryst D61:651–657. 10.1107/S0907444905007808 PMC7159505 15930615

[45] Gorrec F (2009) The MORPHEUS protein crystallization screen. J Appl Cryst 42:1035–1042. 22477774 10.1107/S0021889809042022 PMC3246824

[46] Winter G (2010) xia2: an expert system for macromolecular crystallography data reduction. J. Appl. Crystallogr. 43:186–190.

[47] Waterman DG , Winter G , Parkhurst JM , Fuentes‐Montero LG , Hattne J , Brewster A , Sauter NK , Evans G , Rosenstrom P (2013) The DIALS framework for integration software. CCP4 Newslett. Protein Crystallogr 49:13–15.

[48] Evans P (2006) Scaling and assessment of data quality. Acta Crystallographica Section D: Biological Crystallography 62:72–82. 16369096 10.1107/S0907444905036693

[49] McCoy AJ , Grosse‐Kunstleve RW , Adams PD , Winn MD , Storoni LC , Read RJ (2007) Phaser crystallographic software. J Appl Cryst 40:658–674. 19461840 10.1107/S0021889807021206 PMC2483472

[50] Emsley P , Cowtan K (2004) Coot: model‐building tools for molecular graphics. Acta Crystallogr D60:2126–2132. 10.1107/S0907444904019158 15572765

[51] Smart OS , Womack TO , Flensburg C , Keller P , Paciorek W , Sharff A , Vonrhein C , Bricogne G (2012) Exploiting structure similarity in refinement: automated NCS and target‐structure restraints in BUSTER. Acta Cryst D68:368–380. 10.1107/S0907444911056058 PMC3322596 22505257

[52] Chen VB , Arendall WB , Headd JJ , Keedy DA , Immormino RM , Kapral GJ , Murray LW , Richardson JS , Richardson DC (2010) MolProbity: all‐atom structure validation for macromolecular crystallography. Acta Cryst D66:12–21. 10.1107/S0907444909042073 PMC2803126 20057044

[53] Sievers F , Wilm A , Dineen D , Gibson TJ , Karplus K , Li W , Lopez R , McWilliam H , Remmert M , Söding J (2011) Fast, scalable generation of high‐quality protein multiple sequence alignments using Clustal omega. Mol Syst Biol 7:539. 21988835 10.1038/msb.2011.75 PMC3261699

