# Supplementary figures and images for: Insights from the crystal structure of the chicken CREB3 bZIP suggest that members of the CREB3 subfamily transcription factors may be activated in response to oxidative stress
Source: Protein Sci. 2019 Feb 6;28(4):779–87. doi: 10.1002/pro.3573 (PMC6423718; doi:10.1002/pro.3573)

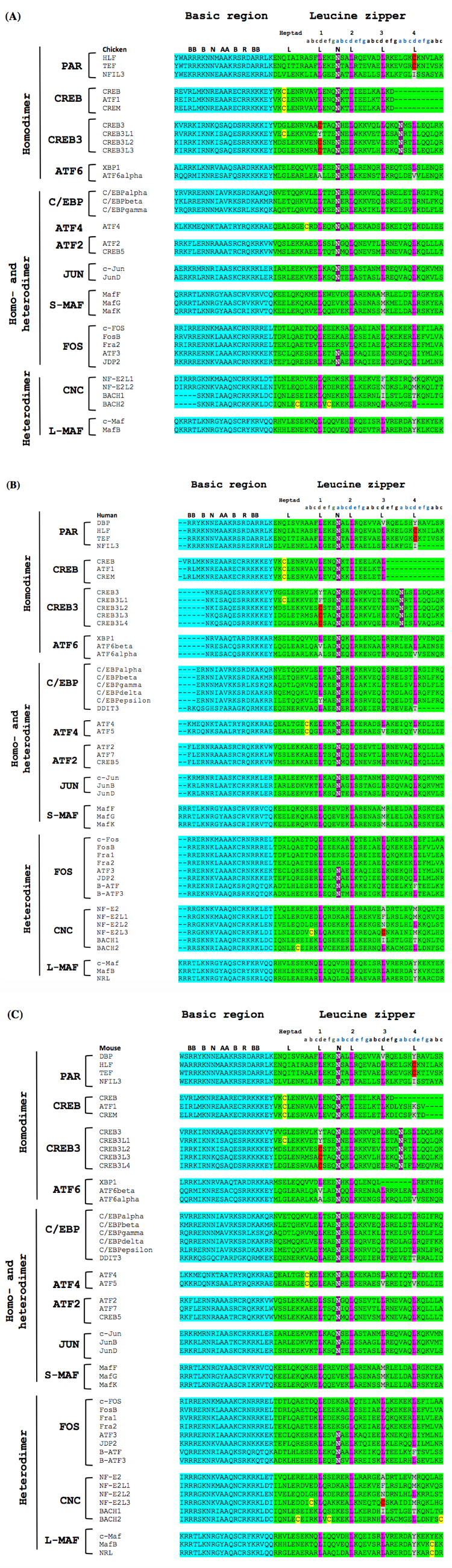

Supplement: Supplementary file 1 — Figure S1 The bZIP regions of chicken (A), human (B), and mouse (C) bZIP transcription factors were aligned using Clustal. The consensus sequence is shown above the sequence alignment (B = any basic residue; N = asparagine; A = alanine; R = arginine; L = leucine). As described in C. Vinson et al. (2002), proteins were placed into 12 groups based on predicted dimerization properties. The basic and leucine zipper regions are highlighted in cyan and green, respectively. Within the leucine zipper region, leucine residues at position d of the heptads are highlighted in magenta; cysteine residues at position d of the heptads are highlighted in red; any residue other than leucine or cysteine at position d of the heptads is highlighted in gray; cysteine residues at any position other than d are highlighted in yellow; and asparagine residues that occupy position a of the second and fourth heptads are highlighted. [file PRO-28-779-s001.tiff]

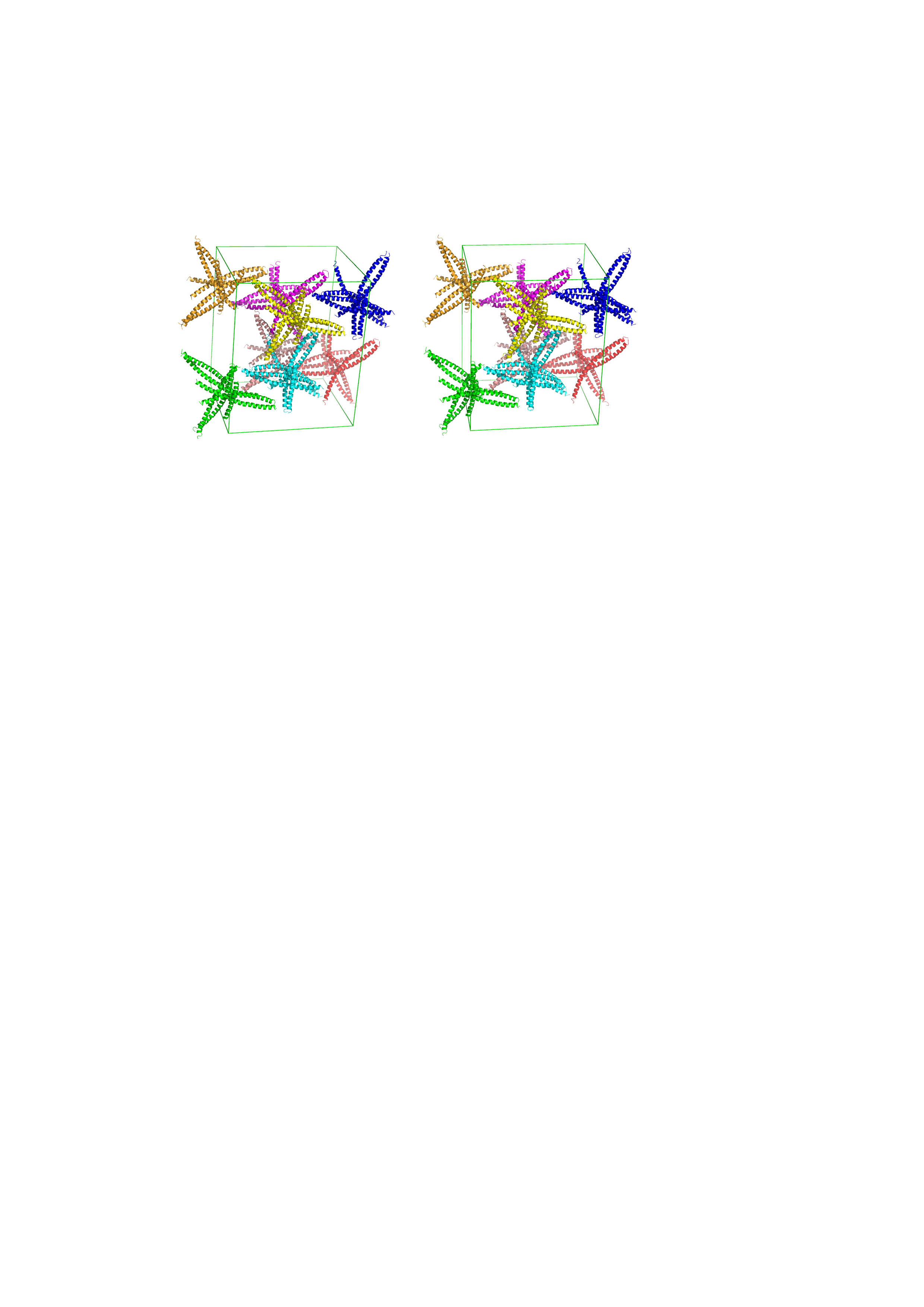

Supplement: Supplementary file 2 — Figure S2 Stereo image of the unit cell and the contents of the asymmetric units. The bZIP domain molecules in each asymmetric unit are colored identically. [file PRO-28-779-s002.tif]
